# Comparative genome analysis of colistin-resistant *Escherichia coli* harboring *mcr* isolated from rural community residents in Ecuador and Vietnam

**DOI:** 10.1371/journal.pone.0293940

**Published:** 2023-11-02

**Authors:** Hoa Thi Thanh Hoang, Mayumi Yamamoto, Manuel Calvopina, Carlos Bastidas-Caldes, Diep Thi Khong, Thang Nam Nguyen, Ryuji Kawahara, Takahiro Yamaguchi, Yoshimasa Yamamoto

**Affiliations:** 1 United Graduate School of Drug Discovery and Medical Information Sciences, Gifu University, Gifu, Japan; 2 Health Administration Center, Gifu University, Gifu, Japan; 3 One Health Research Group, Universidad De Las Americas, Quito, Ecuador; 4 Center for Medical and Pharmaceutical Research and Service, Thai Binh University of Medicine and Pharmacy, Thai Binh, Vietnam; 5 Department of Microbiology, Osaka Institute of Public Health, Osaka, Japan; Zhejiang University, CHINA

## Abstract

The spread of colistin-resistant bacteria among rural community residents of low- and middle-income countries is a major threat to community health. Although the mechanism of the spread of colistin-resistant bacteria in communities is unknown, geographic and regional characteristics may influence it. To elucidate the spread mechanism of colistin-resistant bacteria, we analyzed the genomes of colistin-resistant *Escherichia coli* isolated from Vietnam and Ecuador residents, which are geographically and socially different. Stool specimens of 139 and 98 healthy residents from Ecuador and Vietnam rural communities, respectively, were analyzed for colistin-resistant *E*. *coli* with *mcr*. Its prevalence in the residents of all the communities assessed was high and approximately equal in both countries: 71.8% in Ecuador and 69.4% in Vietnam. A phylogenetic tree analysis revealed that the sequence type of colistin-resistant *E*. *coli* was diverse and the major sequence types were different between the two countries. The location of *mcr* in the isolates showed that the proportion of chromosomal *mcr* was 35.1% and 8.5% in the Vietnam and Ecuador isolates, respectively. Most of these chromosomal *mcr* genes (75%–76%) had an intact *mcr*-transposon Tn*6330*. Contrastingly, the replicon types of the *mcr*-carrying-plasmids were diverse in both countries, but almost all belonged to IncI2 in Ecuador and IncX1/X4 in Vietnam. Approximately 26%–45% of these *mcr*-plasmids had other resistance genes, which also varied between countries. These results suggest that although the overall profile of the colistin-resistant *E*. *coli* isolates is diverse in these countries, the phylogenesis of the isolates and *mcr*-carrying plasmids has regional characteristics. Although the contributing factors are not clear, it is obvious that the overall profile of colistin-resistant bacteria dissemination varies between countries. Such different epidemic patterns are important for establishing country-specific countermeasures against colistin-resistant bacteria.

## Introduction

The recent emergence and spread of carbapenem-resistant Gram-negative bacteria poses a major threat to human health [1]. Despite their toxicity, polymyxins are used as a valuable therapy for treating infections caused by these bacteria [2, 3]. Colistin (polymyxin E) belongs to the family polymyxin and has been available in clinical practice since the 1960s; so far, colistin is still the primary treatment for infections caused by carbapenem-resistant Gram-negative pathogens [4]. Colistin resistance was known by chromosomal mutations relating to the structural alteration of lipid A in the lipopolysaccharide (LPS), a binding target of colistin [5, 6]. Common mutations that occurred with colistin resistance in *Escherichia coli* were reported in associated genes like *pmr*, *pho*, and *mgrB*. Recently, the mobile resistance genes known as *mcr* genes, which encode a phosphoethanolamine transferase, have been identified. This enzyme modifies the lipid A structure, subsequently reducing the binding affinity of colistin to LPS [6]. It has been identified as a primary mechanism for colistin resistance due to its mobility. Thus far, at least ten *mcr* variants from *mcr-1* to *mcr-10* have been described globally [7, 8]. It is widely accepted that the major mechanism of the horizontal spread of colistin-resistant bacteria is the transfer of the mobile *mcr* genes among bacteria [9, 10].

Colistin-resistant *mcr*-mediated *E*. *coli* is present globally, especially in Asia, Europe, and South America [11, 12]. In a previous study, we found that the prevalence of colistin-resistant *E*. *coli* carrying *mcr-1* isolated from the residents of a community of Vietnam was 69.3% [13]. However, knowledge regarding the spread of colistin-resistant bacteria with *mcr* in other communities/regions is limited; particularly, studies comparing the effects of geographical and cultural backgrounds of residents of different countries on the spread of these pathogens are lacking.

Colistin-resistant *E*. *coli* carrying *mcr* has also been found in high frequencies in domestic livestock in local communities of Vietnam, as reported in our previous studies [10, 14]. In another study, the prevalence of *mcr-1* and *mcr-3* detected in human and animal stools in a rural community of Vietnam were higher than that in previous reports [15]. The same situation was observed in a rural small-scale farm in Ecuador, where *mcr*-mediated colistin-resistant *E*. *coli* was widespread [16]. Due to the high prevalence of colistin-resistant *E*. *coli* carrying *mcr* in livestock as well as the possibility of horizontal transfer among various hosts, there is a high risk of the spread of resistant bacteria in the human community, given that animals are used for food production [17]. Such a spread among communities and regions may be influenced by the people’s lifestyles, which may also affect colistin-resistant bacteria epidemic patterns. Ecuador is located in Latin America with an upper middle-income level, whereas Vietnam is a lower middle-income country in Southeast Asia [18, 19]. Although both Ecuador and Vietnam are tropical countries with characteristics of high temperature and humidity, the average temperature in Vietnam was observed to be higher than in Ecuador. In addition, the primary agricultural practices of the two countries differ significantly. These two countries are also different regarding religions, while 85.6% of Ecuador residents belong to Christianity, 73.2% of Vietnam residents are nondenominational [20]. Therefore, it is important to examine the dissemination of colistin-resistant bacteria in geographically and culturally different regions to elucidate the mechanism underlying the epidemic spread of colistin-resistant bacteria. Therefore, this study aimed to assess the spread of colistin-resistant bacteria in Vietnam and Ecuador using a comparative genome analysis approach.

## Materials and methods

### Specimen collection

Stool samples provided by human residents of the communities were used as specimens in the study. Specimen collection was conducted in one community in Vietnam from November 1, 2017 to February 28, 2018 and in two communities in Ecuador from February 15 to March 8, 2019. These communities were relatively small rural villages. The major exclusion criterion for participants was antibiotic use in the last six months. A total of 237 healthy residents of Nguyen Xa in the Thai Binh province, Vietnam, Santo Domingo in the Pacific Coastal, and Puyo in the Amazon of Ecuador were enrolled. The participant characteristics are shown in Table 1. One stool specimen was obtained from each resident. Specimen collection and bacterial isolation in Vietnam have previously been reported [13]; the isolated bacteria were used for analysis in this study.

**Table 1:** Participant characteristics and detection of colistin-resistant *E*. *coli* carrying *mcr* in their stool specimens.

Country	Area	Community	Number of participants	Age		Sex	Number of residents carrying colistin-resistant *mcr-E*. *coli* (% positive*)*
				Median	Range	Male (%)	
Ecuador	La Costa	Santo Domingo	72	28	7–78	45.8	53 (73.6)
	El Oriente	Puyo	67	14	4–67	40	47 (70.1)
Vietnam^a^	Red river delta	Nguyen Xa	98	46	2–81	44.9	68 (69.4)

^a^Data from the work of Yamamoto et al. [13]

### Isolation of colistin-resistant *E*. *coli*

The stool specimens were directly inoculated onto selective agar medium CHROMagar^™^ COL-APSE plates (CHROMagar, Paris, France) for isolation of the colistin-resistant Gram-negative bacteria. The resulting *E*. *coli*-like colonies, which showed pink to red color on the agar, were isolated.

### Characterization of the isolates

The colistin-resistant *E*. *coli* isolates were identified via biochemical test using the analytical profile index (API) 20E system (bioMerieux, Marcy-l’Étoile, France) and matrix-assisted laser desorption/ionization time-of-flight mass spectrometry (MALDI-TOF/MS) (bioMérieux Japan, Tokyo, Japan).

### Multiplex PCR for colistin-resistant genes

Bacterial DNA was extracted by boiling the bacterial suspension for 10 min in tris (hydroxymethyl) aminoethane-ethylenediaminetetraacetic acid (EDTA) buffer. The PCR screening for *mcr-1* to *mcr-5* was performed using the QIAGEN Multiplex PCR Plus kit (Qiagen, Hilden, Germany). The primers, PCR conditions, and electrophoresis was done according to a method described previously [21].

### Antimicrobial susceptibility testing

The colistin minimum inhibitory concentration (MIC) of colistin-resistant *E*. *coli* was measured using an agar dilution method as previously described [22]. MICs ≥2 μg/mL were interpreted as resistance to colistin according to European Committee on Antimicrobial Susceptibility Testing (EUCAST) 2021 guidelines (https://www.eucast.org/fileadmin/src/media/PDFs/EUCAST_files/Guidance_documents/Colistin_guidance_2021.pdf).

### Whole-genome DNA sequencing and analysis

#### DNA extraction

The genomic DNA of the isolates was extracted using NucleoBond HMW DNA (Macherey-Nagel, Düren, Germany) according to the manufacturer’s protocol. The DNA was quantified using Qubit Double-Stranded DNA High-Sensitivity Assay Kits (Thermo Fisher Scientific, Waltham, MA, USA) and quality was assessed using a NanoDrop (Thermo Fisher Scientific). The A_260/280_ and A_260/230_ ratios of the DNA samples were within the range mentioned in the manufacturer’s instructions for genome sequencing.

#### Library preparation and sequencing

Whole genome sequences were obtained using the DNBSEQ-G400FAST (MGI Tech, Shenzhen, China) and MinION Mk1C sequencer (Oxford Nanopore Technologies, London, UK). Short-read sequencing using the DNBSEQ-G400RS High-throughput Sequencing Set (MGI Tech) was performed by a commercial vendor (Genome-Lead Co., Kagawa, Japan). For the long-read sequencing, which was carried out using a MinION, the high molecular weight DNA of each isolate was barcoded using the Rapid Barcoding Kit (Oxford Nanopore Technologies) and was pooled into one PCR tube. To prepare the library, sequencing adapters were attached to the DNA sequences. The MinION flow cell (R9.4.1) was primed and loaded with the prepared library for a 24-h run on the MinION Mk1C.

#### *De novo* assembly

To obtain the complete genome of all tested isolates, a *de novo* hybrid assembly of both short- and long-reads was conducted using Unicycler 0.4.8, with default settings, on a supercomputer system and CLC Genomics Workbench 21.0.3. The short-read sequence was trimmed, and its quality was checked using fastp 0.20.1 and FastQC 0.11.9. The quality of long reads was verified using NanoPlot (http://nanoplot.bioinf.be/). In Unicycler, SPAdes v3.13.1 (with the maximum k-mer 127), Miniasm, Racon v.1.4.3, bowtie2 v.2.3.5, and SAMtools v.1.9 were run for assembly, and the complete bacterial genome was polished using Pilon v.1.23. Using the CLC Genomics Workbench, *de novo* assembly of long reads was performed in the Long Read Support (beta) plugin, followed by polishing with short reads using the Polish with Reads (beta) tool. In this study, we obtained a total of 84 complete *E*. *coli* genomes (S1 and S2 Tables).

#### Annotations

The complete assembled sequences were annotated by uploading the FASTA files to the DNA Data Bank of Japan (DDBJ) Fast Annotation and Submission Tool (DFAST) v.1.6.0. (https://dfast.ddbj.nig.ac.jp/dfc/) and were confirmed using the ResFinder 4.1 database (https://cge.food.dtu.dk/services/ResFinder/); they were then deeply analyzed using Genious Prime v.2022.0.1. In addition, the *E*. *coli* plasmid replicons were detected using PlasmidFinder 2.1 (https://cge.food.dtu.dk/services/PlasmidFinder/) with a 95% and 60% threshold for minimum identification and minimum coverage, respectively.

#### Genome comparison

The similarity between a central reference sequence and others in this study was illustrated using BLAST Ring Image Generator (BRIG) 0.95 (http://brig.sourceforge.net).

#### Phylogenetic tree generation

The phylogenetic tree of the isolates was based on the single nucleotide polymorphism (SNPs) data generated using PARsnp version 1.7.4, run on multi-MUM as well as libMUSCLE aligner (https://github.com/marbl/parsnp), and constructed using iTOL (https://itol.embl.de). The initial tree was generated automatically (by default).

### Statistics

The data of the number of residents carrying *mcr-E*. *coli*, the *mcr-1* location, types of *mcr-1*-transposon structures, dissemination of Inc-types of *mcr*-plasmids, and plasmids with antimicrobial-resistant (AMR) genes were compared among three groups of areas via the chi-square test and descriptive statistics using the statistical package for the social sciences (SPSS) version 20.0 software (IBM Corporation, Armonk, NY, USA). A *p*-value of <0.05 was considered statistically significant.

## Results

### Prevalence of colistin-resistant *E*. *coli* carrying *mcr* in stool specimens of the residents

Results of the prevalence of *mcr*-positive colistin-resistant *E*. *coli*, which showed colistin MIC ≥4 μg/mL in healthy residents in Vietnam and Ecuador, are shown in Table 1. The prevalence of colistin-resistant *E*. *coli* with *mcr-1* in the three communities of the two countries was similar, ranging from 69%–73%, with no significant difference. Notably, among selected isolates for whole genome sequencing, all 84 isolates carrying the *mcr-1*.*1* gene, as shown in S1 and S2 Tables.

### Phylogenetic analysis of colistin-resistant *E*. *coli* isolates among the communities

**Fig 1:**
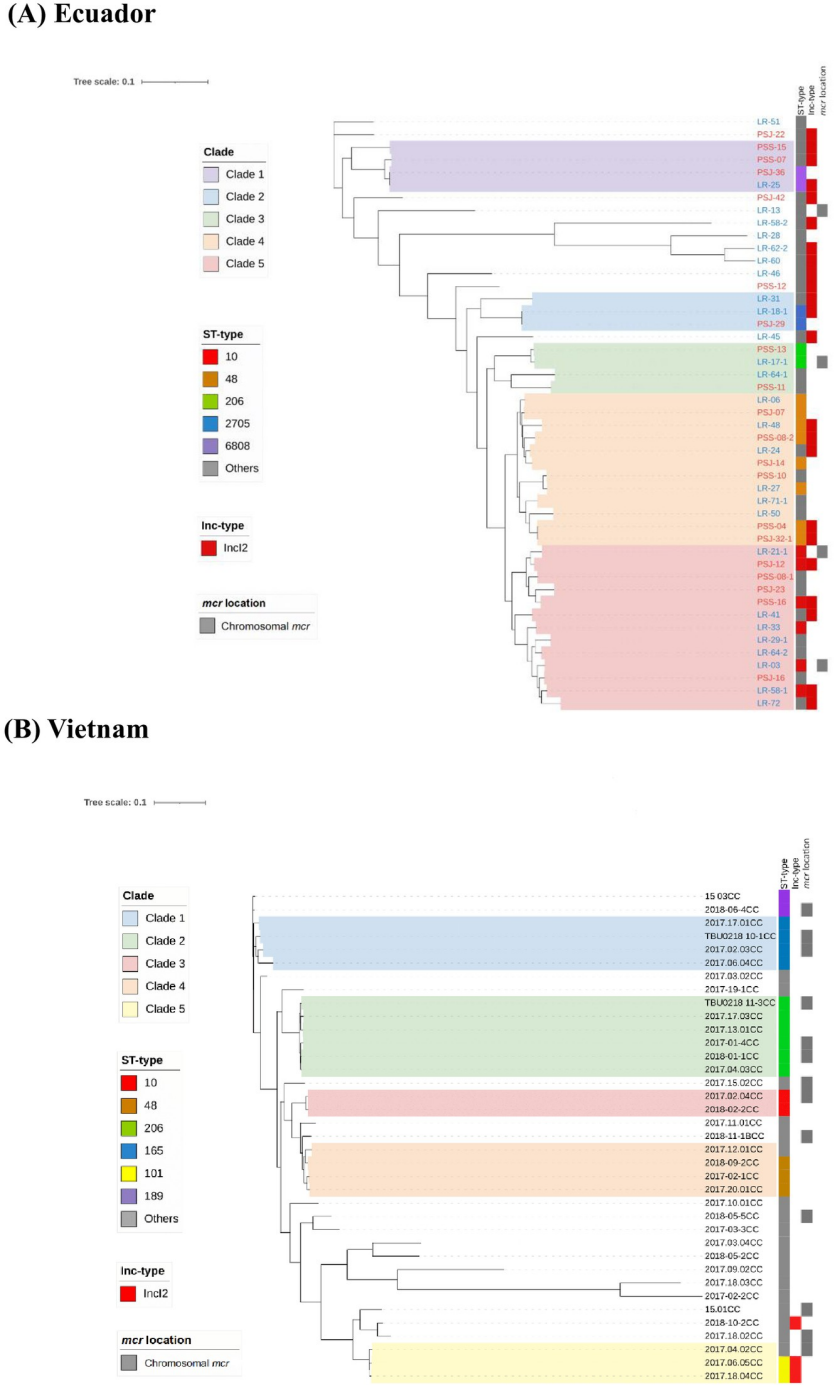
The SNP phylogenetic tree of colistin-resistant *E*. *coli* isolates obtained from residents in (A) Ecuador and (B) Vietnam. The scale bar represents the nucleotide substitutions per site. The isolate IDs shown in blue and red in (A) indicate those isolated from Santo Domingo and Puyo, respectively. The Inc-type legend indicates isolates carrying the IncI2 plasmid with *mcr-1*. The *mcr* location legend indicates the presence of chromosomal *mcr*.

In this study, approximately half of the isolates from both countries, including 47 of 100 *mcr-E*. *coli* isolates in Ecuador and 37 of 68 *mcr-E*. *coli* isolates in Vietnam were randomly selected for sequencing. The complete genome was obtained for all 84 isolates that were sequenced (S1 and S2 Tables). The phylogenetic trees of colistin-resistant *mcr-E*. *coli* isolates from both countries were then generated using core-genome SNPs (Fig 1).

The isolates obtained from Ecuador residents were allocated to 30 sequence types (ST-types). Among them, two ST-types, ST48 and ST10, were dominant and three—ST206, ST2705, and ST6808—were more prevalent than the other ST-types, which appeared only once among the isolates. Based on the ST-types and the similarity among homologous sequences, the isolates were clustered into five major clades (Fig 1A). The first clade (clade 1) included four *E*. *coli* isolates; three of them were isolated in Puyo and one was from Santo Domingo with two ST6808 isolates. The second and third clades with three and four isolates mainly included ST2705 and ST206, respectively. The fourth and fifth clades represented two major branches among the Ecuador isolates, which comprised 12 and 13 strains with predominantly ST48 and ST10, respectively. As shown in Fig 1A, the colistin-resistant *E*. *coli* isolates from samples from both Santo Domingo and Puyo were diverse; however, 48.9% of them carried the IncI2 plasmid with *mcr-1*.

In Vietnam, 21 major ST-types, including ST165, ST206, ST48, and ST10, were detected among the 37 isolates, as in Ecuador. Five clades were clustered from these isolates. In contrast to Ecuador samples, ST165, ST206, and ST10 were the only STs in clades 1, 2, and 3, respectively. The fourth clade included three ST48 and the fifth clade included two ST101 isolates. Only three isolates (8.1%) carried the *mcr*-IncI2 plasmid among the Vietnam isolates (Fig 1B).

The phylogenetic trees of all the isolates obtained from Ecuador and Vietnam samples were analyzed together. As shown in S1 Fig, the colistin-resistant *E*. *coli* isolates from both countries were phylogenetically diverse and covered most branches. For instance, ST10, ST48, and ST206 were found in large numbers in both Ecuador and Vietnam samples.

### *mcr* location

Since a high chromosomal *mcr* proportion of 35.1% was observed in Vietnam isolates [23], we used genome analysis to examine whether a similar chromosomal *mcr* prevalence also existed in Ecuador isolates. As shown in Table 2, only 14.8% of the Santo Domingo isolates carried *mcr-1* on their chromosomes. All the Puyo isolates only harbored *mcr-1* on the plasmid, and none of the isolates possessed chromosomal *mcr*. Thus, the chromosomal *mcr* proportion of the *mcr*-*E*. *coli* isolates in Ecuador was significantly lower than that in Vietnam (*p* = 0.006). However, a difference in chromosomal *mcr* ratios was not observed between communities within Ecuador (*p* = 0.204).

**Table 2:** Location and structure of the *mcr-1*-transposon.

Country	Community	No. of isolates tested	*mcr-1* location	Number (%)	*mcr-1*-transposon structure^b^		
					Number of isolates (%)		
					A	B	C
Ecuador	Santo Domingo	27	Chromosome	4 (14.8)	3 (75)	0	1 (25)
			Plasmid	23 (85.2)	9 (39.1)	2 (8.7)	12 (52.2)
	Puyo	20	Chromosome	0	0	0	0
			Plasmid	20 (100)	9 (45)	1 (5)	10 (50)
Vietnam	Nguyen Xa	37^a^	Chromosome	13 (35.1)	10 (76.9)	3 (23.1)	0
			Plasmid	24 (64.9)	0	6 (25)	18 (75)

^a^Part of the data is quoted from the work of Yamaguchi et al. [23].

^b^A: IS*Apl1*-*mcr1*-*PAP2*-IS*Apl1*; B: IS*Apl1*-*mcr1*-*PAP2*; C: *mcr1*-*PAP2*

Contrastingly, no association was observed among the ST-types harboring chromosomal *mcr* in samples from both countries (Fig 1).

### Transposon structure of *mcr*

Transposon structure is important for *mcr* transfer and stability. We compared the *mcr* transposon structures of all isolates. Results showed that the structure of the *mcr-*transposon was different between the chromosome and the plasmid of the isolates among all three communities. In the isolates from both Santo Domingo in Ecuador and Nguyen Xa in Vietnam, the intact *mcr-*transposon Tn*6330* structure (type A), IS*Apl1-mcr1-PAP2-*IS*Apl1*, was dominantly located on the chromosome (Table 2). In contrast, the prevalence of the incomplete *mcr*-transposon structures, such as type B, IS*Apl1-mcr1-PAP2*, and type C, *mcr1-PAP2*, was low on the chromosome. There was no isolate with type B in Santo Domingo samples and no isolate with type C in Nguyen Xa samples.

The *mcr-*transposon structure on the plasmids also differed between Ecuador and Vietnam isolates. As shown in Table 2, all the Vietnam isolates possessed plasmids carrying an incomplete *mcr-*transposon, such as type B or C. There was no plasmid with intact *mcr-*transposon type A. In contrast, many plasmids (39%–45%) in the isolates from both communities in Ecuador had an intact *mcr-*transposon type A. However, more than half of the *mcr-*transposons were type B, lacking a partial insertion sequence, IS*Apl1*, or type C, which was completely lacking.

For further analysis of the *mcr*-transposon genetic characteristics, the genetic environment around the *mcr-*transposon on the chromosome was assessed. Of the 17 isolates with chromosomal *mcr*, 16 isolates carrying *mcr*-transposons with insertion sequence (IS), type A and B, were analyzed for their chromosomal insertion sites. The results showed that the insertion sites of the *mcr*-transposon on the chromosomes of all these isolates were surrounded by AT-rich regions. A representative genetic environment map of the *mcr*-transposon on the 2018-11-1BCC chromosome of Vietnam is shown in S2 Fig.

### *mcr*-plasmid incompatibility (Inc)-type

The Inc-type of the *mcr*-plasmid is important for plasmid transfer and spread. The regional Inc-type of *mcr*-carrying plasmids, which are the main cause of the spread of *mcr*-carrying colistin-resistant bacteria, was assessed. As shown in Table 3, among the three communities, the Inc-type of *mcr*-carrying plasmids was diverse and disseminated differently; moreover, the major *mcr*-carrying plasmid type differed between Ecuador and Vietnam isolates. I2-plasmids were significantly more frequent among the Ecuador isolates, at 50%–56%. In contrast, the P1 and X1/X4 plasmids were dominant in the Vietnam isolates, at 25%–29%. The hybrid plasmids carrying *mcr* were also dominant, ranging from 13%–35%, with no significant differences among the communities. Regarding the hybrid types, FIA/HIA/HIB was dominant in the Puyo isolates, while HI2/HI2A was dominant in the Nguyen Xa isolates (Table 4).

**Table 3:** Inc-type of plasmid carrying *mcr-1*.

Country	Community	No. of isolates tested	No. of plasmids	Plasmid Inc-type						
				P1	I2	N	FIB/ FII	X1/ X4	Y	Hybrid
Ecuador	Santo Domingo	27	23	3 (13%)	13 (56.5%)	1 (4.4%)	2 (8.7%)	1 (4.4%)	0	3 (13.0%)
	Puyo	20	20	0	10 (50%)	2 (10%)	0	1 (5%)	0	7 (35%)
Vietnam	Nguyen Xa	37	24^a^	6 (25%)	3 (12.5%)	0	1 (4.2%)	7 (29.2%)	2 (8.3%)	5 (20.8%)

^a^Part of the data is quoted from the work of Yamaguchi et al. [23].

**Table 4:** Inc-type of hybrid plasmids carrying *mcr-1*.

Country	Community	No. of plasmids	Inc-type					
			HI2/HI2A	HI2/HI2A/N	FIA/HIA/HIB	FIA/HIA/HIB/p0111	FIA/FIB	HI1A/HI1B
Ecuador	Santo Domingo	3	0	0	2	0	1	0
	Puyo	7	0	0	5	1	0	1
Vietnam	Nguyen Xa	5	3	1	1	0	0	0

**Fig 2:**
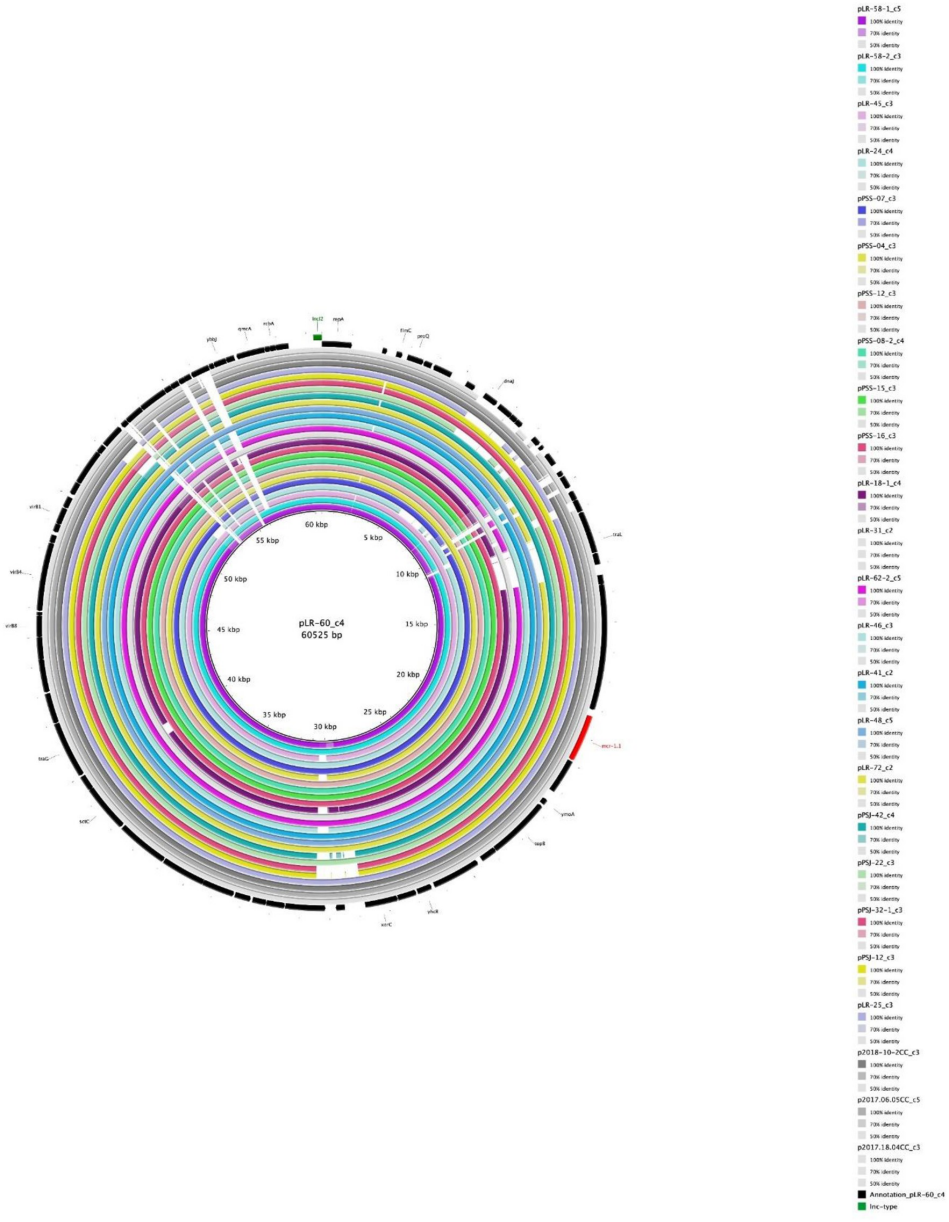
Comparative genomics of the IncI2 plasmids with *mcr-1*.*1*. The gray color indicates a Vietnam isolate. The other colors indicate an Ecuador isolate.

The structures of I2-plasmids in both Ecuador and Vietnam isolates were compared; the results showed that the size and constituent genes were similar among these plasmids (Fig 2). Similarly, the sequences of other plasmid Inc-types, including X1, X4, and P1 *mcr*-plasmids, were compared. As shown in S3–S5 Figs, these plasmid Inc-types were also similar across communities and countries.

### AMR genes on *mcr*-plasmids

Among the plasmids carrying *mcr-1* from isolates, the presence of other AMR genes was assessed in Ecuador and Vietnam. As shown in Table 5 (refer to S1 and S2 Tables for details), the percentage of *mcr*-plasmids carrying no other AMR genes regardless of which community the isolate was from was higher than multi-AMR genes carrying plasmids, with 60%, 54.2%, and 73.9% in Puyo, Nguyen Xa, and Santo Domingo, respectively. However, *mcr*-carrying hybrid plasmids, which amalgamate two or more distinct Inc-type plasmid, in the isolates from both countries carried several AMR genes. As shown in Table 6, the hybrid plasmids of Puyo isolates mainly possessed genes encoding resistance to sulfonamide, streptomycin/spectinomycin, trimethoprim, tetracycline, beta-lactam, aminoglycoside, and chloramphenicol, whereas those of Vietnam isolates mainly possessed aminoglycoside resistance genes.

**Table 5:** Antimicrobial resistance (AMR) genes on plasmids carrying *mcr-1*.

Country	Community	No. of plasmids	With no AMR gene	With multi-AMR genes
Ecuador	Santo Domingo	23	17 (73.9%)	6 (26.1%)
	Puyo	20	12 (60%)	8 (40%)
Vietnam	Nguyen Xa	24	13 (54.2%)	11 (45.8%)

**Table 6:** Profile of AMR genes on plasmids carrying *mcr-1*.

Antimicrobials	AMR gene	Ecuador					Vietnam			
		Santo Domingo			Puyo		Nguyen Xa			
		Inc-type								
		P1 (n = 1)*	FII (n = 2)	Hybrid (n = 3)	X1/4 (n = 1)	Hybrid (n = 7)	P1 (n = 3)	X1/4 (n = 2)	FIB (n = 1)	Hybrid (n = 5)
Sulfonamide	*sul1*									1
	*sul2*	1**		1		1			1	1
	*sul3*			1	1	5			1	3
Streptomycin/spectinomycin	*aadA2*			3		6			1	1
	*aadA5*	1								
Trimethoprim	*dfrA12*			2		4			1	
	*dfrA14*									1
	*dfrA15*									1
	*dfrA17*	1								
Tetracycline	*tet(A)*				1	2			1	2
	*tet(B)*			1		5				
	*tet(M)*			2				2		3
Beta-lactam	*bla*_TEM-1B_			1		3		1	1	1
	*bla*_TEM-106_						3			2
	*bla*_TEM-135_									1
	*bla*_TEM-141_					4				
	*bla*_TEM-176_				1					
	*bla*_TEM-230_									1
	*bla*_CTX-M-55_									1
Fosfomycin	*fosA3*			1						
	*fosA6*		2							
Aminoglycoside	*ant(3’’)-Ia*			2	1	5				2
	*aph(3’)-Ia*				1				1	4
	*aph(3’’)-Ib*					1				6
	*aph(6)-Id*					1				4
	*aac(3)-IId*								1	3
Rifampin	*ARR-2*									1
Chloramphenicol	*cmlA1*			2		5				
Florfenicol/chloramphenicol	*floR*			1	1	3		2	1	4
Quinolone	*qnrB19*				1	2				
	*qnrS1*									1
Lincosamide	*lnu(F)*									1

* Number of *mcr-1*-carrying plasmids harbored other AMR genes with corresponding Inc-type

**Number of plasmids carrying AMR genes.

## Discussion

### Prevalence of colistin-resistant *E*. *coli* carrying *mcr*

This study revealed a high carriage rate of approximately 70% of colistin-resistant *mcr*-*E*. *coli* in the residents of all three communities, Santo Domingo and Puyo in Ecuador and Nguyen Xa in Vietnam. Recent reports by other groups showed the prevalence of *mcr*-*E*. *coli* to be 14%–38% in human stool samples from Vietnam [24, 25] and Bolivia in South America [26]. Although it is lower than the carriage rate observed in the present study, it is presumed that this may be due to differences in the experimental conditions and participants. In particular, the selection medium used for colistin-resistant bacteria may greatly affect the carriage rate of resistant bacteria in the stools. Nevertheless, such a high colistin-resistant *E*. *coli* carriage found among the residents in the study was associated with a high prevalence of colistin-resistant *E*. *coli* in the livestock of the same area [14, 16], which might be relevant with regards to the One Health [27]. The similar levels of colistin-resistant *E*. *coli* in these geographically and culturally different communities/regions indicate that the wide dissemination of colistin-resistant *E*. *coli* carrying *mcr* in communities of low- and middle-income countries is a major threat to global public health.

### Phylogenetic homology of colistin-resistant *E*. *coli* isolates

The phylogenetic homology of colistin-resistant *E*. *coli* disseminated among the communities and countries was assessed using the genome sequences of isolates. The Ecuador and Vietnam isolates were clustered into five major clades. However, the number of isolates in each clade and the distribution of sequence types were different between the two countries. In Ecuador isolates, all the clades included mixed types of STs, but ST48 and ST10 were dominant among them. In addition, the *mcr-1*-carrying IncI2 plasmids were frequent among the isolates and distributed in various clades, as opposed to being concentrated in specific clades. The Ecuador isolates were from two geographically distinct communities within the same country—one in the Pacific coast and the other in the Amazon region—but each isolate did not fall into a specific clade of the community. Furthermore, the *mcr*-carrying plasmid IncI2 was widely distributed without bias in a specific clade. These results indicate that there are inter-regional social interactions between the communities. In contrast, in Vietnam, three of the five clades consisted of a single ST. In addition, the *mcr*-carrying plasmid IncI2 was rare in Vietnam isolates. Thus, although two communities in Ecuador and one community in Vietnam were assessed, the STs included in the clade of each country were different. In Vietnam, the spread by specific STs was dominant over the spread by *mcr*-carrying plasmids. It is unclear whether such differences between the two countries are caused by sociocultural differences or other factors. In this regard, it was reported that ST10, ST48, and ST165 were the common ST-types of colistin-resistant *E*. *coli* with *mcr-1*, and among them, ST10 was the most dominant group and was the main contributor to the dissemination of colistin-resistant *E*. *coli* [28, 29]. Besides relating to the colistin resistance, ST10 was also observed as the most common clone producing many extended-spectrum beta-lactamase (ESBL) types in *E*. *coli*, especially *bla*_CTX-M_ [30–33]. In the present study, ST10 was found in limited frequency in both Vietnam and Ecuador isolates, which confirmed that this strain has spread globally. Conversely, the ST48 clone has recently been identified to carry the *bla*_KPC_ gene, which is associated with carbapenem resistance [34, 35]. The spreading of common ST clones carrying multi-AMR genes poses a significant challenge in controlling the antimicrobial resistance of bacteria.

When we analyzed the ST-type of all the *mcr-E*. *coli* isolates from Ecuador and Vietnam together, at least some phylogenies, including ST10, ST48, and ST206, were observed in both countries. This means that some lineages of *E*. *coli* carrying *mcr* are common host strains worldwide. This observation suggests that the *mcr*-plasmids may have spread to the endemic ST-type *E*. *coli* in these communities. That is, the wide dissemination of colistin-resistant *E*. *coli* in local communities is the result of the spread of *mcr*-carrying plasmids to endemic *E*. *coli* clones rather than the spread of *mcr*-carrying *E*. *coli* clones themselves.

### Location and structure of *mcr*-transposon

The analysis of *mcr* location in the isolates from both Vietnam and Ecuador showed that the prevalence of chromosomal *mcr* in Ecuador was significantly lower than that in Vietnam. In particular, no chromosomal *mcr* was found in the Puyo isolates from Ecuador. Such differences in the chromosomal *mcr* frequency presumably indicate differences in the exposure of these organisms to colistin. The prevalence of chromosomal *mcr* in Vietnam isolates of this study is in line with the previous findings of another group that showed that 26% of Vietnam isolates carried *mcr-1* on their chromosomes [24]. This high prevalence of chromosomal *mcr* indicates the stable colistin resistance of *E*. *coli*. On the contrary, the data on *mcr* location in *E*. *coli* isolated from humans and animals in Ecuador is limited. In a study on characteristics of the *mcr* gene among *E*. *coli* from waters and sediments in Ecuador, only 1 of 459 *Enterobacteriaceae* bacteria carried chromosomal *mcr* [36].

The structure of the *mcr*-transposon Tn*6330* has also contributed to the stability and transfer of *mcr* [9]. That is, the *mcr*-transposon is considered to be stabilized by the structure in which insertion sequences (IS*Apl1*) have been lost, although the transfer ability of *mcr* is limited [9, 12]. Interestingly, in the Vietnam isolates, all the *mcr*-transposons on the plasmids lost both or one of the ISs, whereas most of the Ecuador isolates had intact transposons that retained the IS. These results indicate that the *mcr*-transposons on the plasmids of Ecuador isolates may be progressing toward stabilization, whereas the *mcr* on the plasmids of Vietnam isolates are almost stabilized. The cause of these differences is unknown, but it probably depends on how long the community was exposed to colistin, such as the region-wide period of colistin use in domestic livestock. Regarding a report on antibiotic use and resistance in Vietnam, antibiotics were frequently used in animals with 70% of pharmaceutical products. Antibiotics were used not only in land-based livestock but also in aquaculture. In particular, colistin contributed to 5% of antibiotics used [37]. Noteworthy, in the results of an updated study about colistin-based drug (CBD) used in chicken farms in Nguyen Xa, Vietnam, more than half of local farmers preferred using CBD than other antibiotics [38]. Whereas the data on the consumption of antibiotics used in humans and animals was lacking in Ecuador [39]. The ban on colistin for use in animal husbandry has been implemented in Ecuador since 2019, the effectiveness of this ban on colistin in Ecuador was discussed [40, 41]. In other words, colistin use in Vietnam may have been prevalent for a longer period than in Ecuador. This may also be the reason in the case of chromosomal *mcr*.

The chromosomal insertion site of the *mcr*-transposon is a crucial factor related to the *mcr-1* insertion. An AT-rich region has a preference for the insertion site of the *mcr*-transposon, and the transposition forms a 2-bp duplication [42, 43]. The AT-rich region is the site where DNA replication and synthesis are initiated; it is also a low thermodynamic stability region [44]. In this study, it was shown that the IS*Apl1* insertion sites of most of Ecuador and Vietnam isolates were surrounded by AT-rich regions. This finding is consistent with the results of previous studies [42, 43].

### *mcr*-carrying plasmid

The mobile colistin resistance genes, *mcr*, mediated by plasmids is the mechanism of concern leading to the wide dissemination of colistin resistance bacteria worldwide [45]. The *mcr*-carrying plasmids are characterized by the Inc-type, and the main Inc-type is different depending on the site of isolation. This study showed that the Inc-type of the *mcr*-carrying-plasmids was diverse in both Ecuador and Vietnam isolates, but most of the plasmids belonged to four types—IncI2, IncP1, and IncX1/X4. In particular, the *mcr*-carrying IncI2-plasmid was remarkably prevalent in both Ecuador communities. In this regard, it was established that IncI2, IncX4, and IncHI2 contributed to more than 90% of the published *mcr-1*-carrying-plasmid types and were the dominant types [12, 28]. IncI-plasmids, including IncI2, are known to have low copy numbers; the host range is narrow [46, 47], and they can transfer among different bacteria within the *Enterobacteriaceae* family, with *E*. *coli* as the predominant carrier [48]. Therefore, the dominance of IncI2 in *mcr*-carrying plasmids in Ecuador is likely a dominant factor contributing to the transfer of *mcr*. In contrast, the X1/X4 Inc-types were preferentially identified among the Vietnam isolates. Five sub-types of the IncX-plasmids, IncX1–IncX5, have been identified [49]. Within the IncX-group, the IncX4 subtype is the most predominant [50]. Besides IncI2, IncX4 plasmids are also considered to be relevant in the spread of *mcr-1* genes in the family *Enterobacteriaceae* [51, 52]. IncX1/X4 plasmids carrying *mcr* were prevalent in Vietnam isolates, unlike IncI2 in Ecuador isolates, indicating that the Inc-type plasmids involved in community *mcr* transmission were different and distinctive among communities.

The results of the comparative analysis of *mcr-*plasmids between isolates from both countries showed that the structures of *mcr*-carrying-IncI2-plasmids in both Ecuador and Vietnam isolates were almost the same in size and constituent genes. Similar results were also obtained with other Inc-type plasmids carrying *mcr*. Although it is known that the same Inc-types of *mcr*-plasmids carried by different strains are similar [53, 54], this is the first report confirming this result among strains collected across countries. This suggests that *mcr*-carrying plasmids, such as the *mcr*-IncI2 plasmid, are spreading across communities and even countries while transferring among different phylogenetic host bacteria.

### AMR genes on plasmids carrying *mcr*

The presence of other AMR genes on *mcr*-plasmids increases the risk of *mcr-*bacteria dissemination among communities. By detecting AMR genes on plasmids carrying *mcr*, we showed that more than half of the *mcr*-plasmids did not carry other resistance genes. These findings are consistent with a previous report that states that the co-existence of other AMR genes with *mcr-1* on the plasmids occurs at a low percentage [55]. Contrastingly, nearly half of the remaining plasmids, especially the hybrid plasmids, carried many AMR genes besides *mcr*. However, the AMR genes carried by the hybrid plasmid differed between Vietnam and Ecuador isolates, indicating regional characteristics. For example, aminoglycoside resistance genes were frequently present in the hybrid plasmids with *mcr* in the Vietnam isolates, whereas trimethoprim, streptomycin/spectinomycin, and chloramphenicol resistance genes were major AMR genes in the Ecuador isolates. The reasons for the differences in the AMR genes carried on *mcr*-plasmids of the isolates between the two countries are unknown, but it seems to be due to differences in the antibiotics frequently used in particular areas. Since the actual situation of antibiotic use in these areas is unknown, it remains a topic for future study. Nevertheless, *mcr*-plasmids carrying AMR genes are a major public health risk.

### Limitations

As this was a cross-sectional study, temporal changes in the prevalence of colistin-resistant *E*. *coli* in the community remain unclear and will be a subject of future studies.

## Conclusions

This study revealed the high and approximately equal prevalence of colistin-resistant *mcr*-carrying *E*. *coli* in Vietnam and Ecuador residents. The proportion of chromosomal *mcr* among the *mcr*-carrying *E*. *coli* isolates was significantly higher in Vietnam than in Ecuador, indicating that the stabilization of colistin resistance is progressing in Vietnam. Phylogenetic analysis revealed a mixture of STs with regional characteristics and common STs to both communities, indicating regional characteristics of the prevalence of resistant bacteria. Furthermore, it was revealed that the major Inc-types contributing to the spread of *mcr* differed between countries. However, these *mcr*-carrying plasmids were almost the same for each Inc-type even in the isolates from different regions and countries. Thus, it was clarified that the prevalence of colistin-resistant *E*. *coli* in society varies depending on the geography of each community and country. Therefore, a detailed analysis of the prevalence of resistant bacteria in individual regions is essential for developing countermeasures against colistin-resistant bacteria.

## Supporting information

S1 Fig The SNP phylogenetic tree of colistin-resistant *E*. *coli* isolates carrying *mcr-1* obtained from Ecuador and Vietnam residents. The scale bar represents the nucleotide substitutions per site. The isolate IDs in red and blue indicate those isolated from Vietnam and Ecuador, respectively.
(TIF)

S2 Fig A) Genetic environment of *mcr-1*-transposon Tn*6330* on the chromosome of the 2018-11-1BCC strain isolated from Nguyen Xa, Vietnam. B) and C) The AT-rich regions (represented by red squares), that surround IS*Apl1* downstream and upstream of Tn*6330*, in the insertion sites were determined.
(TIF)

S3 Fig Comparative genomics of the IncX1 plasmids with *mcr-1*.*1*. The gray color indicates a Vietnam isolate. The purple color indicates an Ecuador isolate.
(TIF)

S4 Fig Comparative genomics of the IncX4 plasmids with *mcr-1*.*1*. The gray color indicates a Vietnam isolate. The purple color indicates an Ecuador isolate.
(TIF)

S5 Fig Comparative genomics of the IncP1 plasmids with *mcr-1*.*1*. The gray color indicates Vietnam isolates. The purple color indicates Ecuador isolates.
(TIF)

S1 Table Genome of the colistin-resistant *E*. *coli* isolates from healthy residents in Ecuador. (ZIP)

S2 Table Genome of the colistin-resistant *E*. *coli* isolates from healthy residents in Vietnam. (ZIP)
