# Supplementary figures and images for: Comparative genome analysis of colistin-resistant Escherichia coli harboring mcr isolated from rural community residents in Ecuador and Vietnam
Source: PLoS One. 2023 Nov 2;18(11):e0293940. doi: 10.1371/journal.pone.0293940 (PMC10621974; doi:10.1371/journal.pone.0293940)

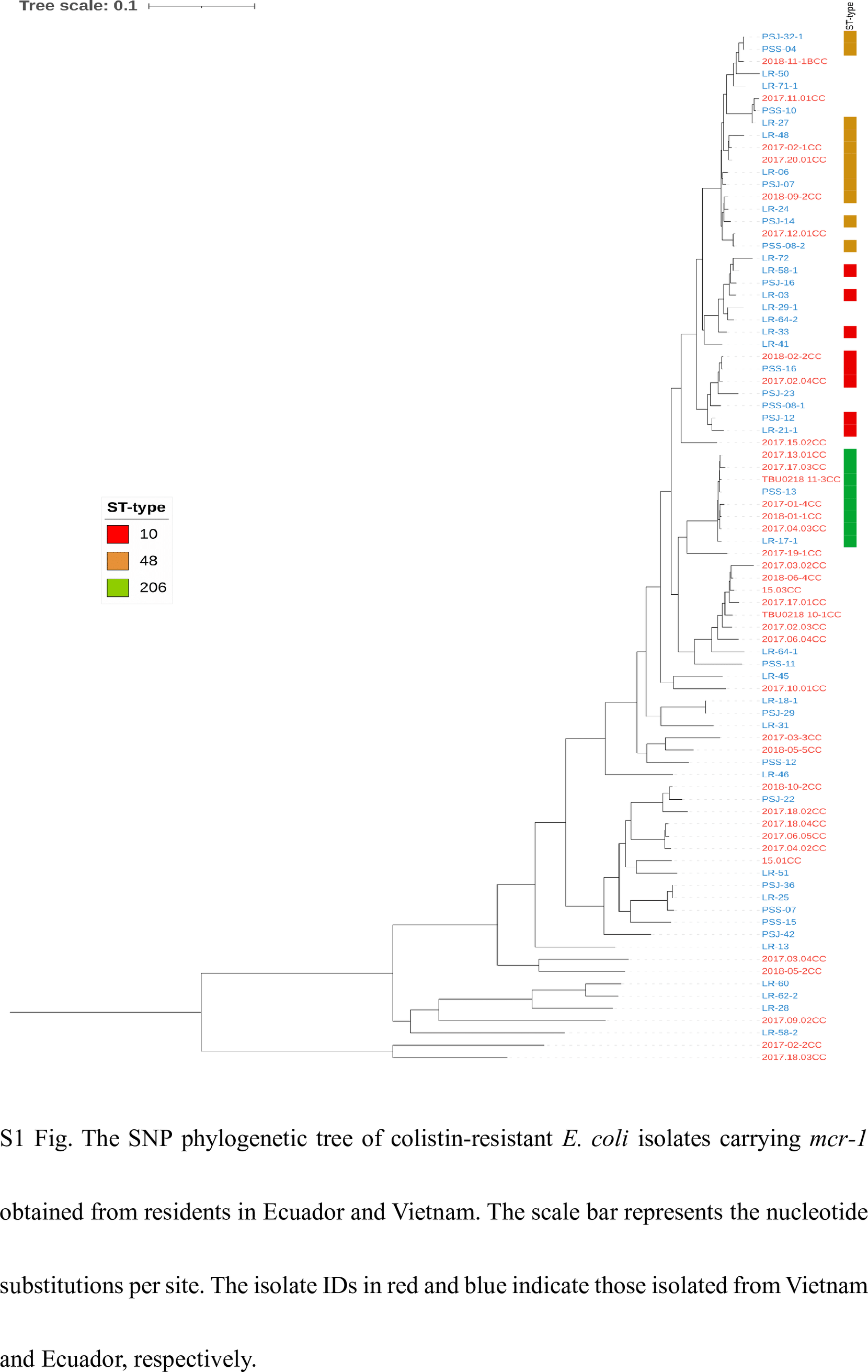

Supplement: S1 Fig — The scale bar represents the nucleotide substitutions per site. The isolate IDs in red and blue indicate those isolated from Vietnam and Ecuador, respectively. (TIF) [file pone.0293940.s001.tif]

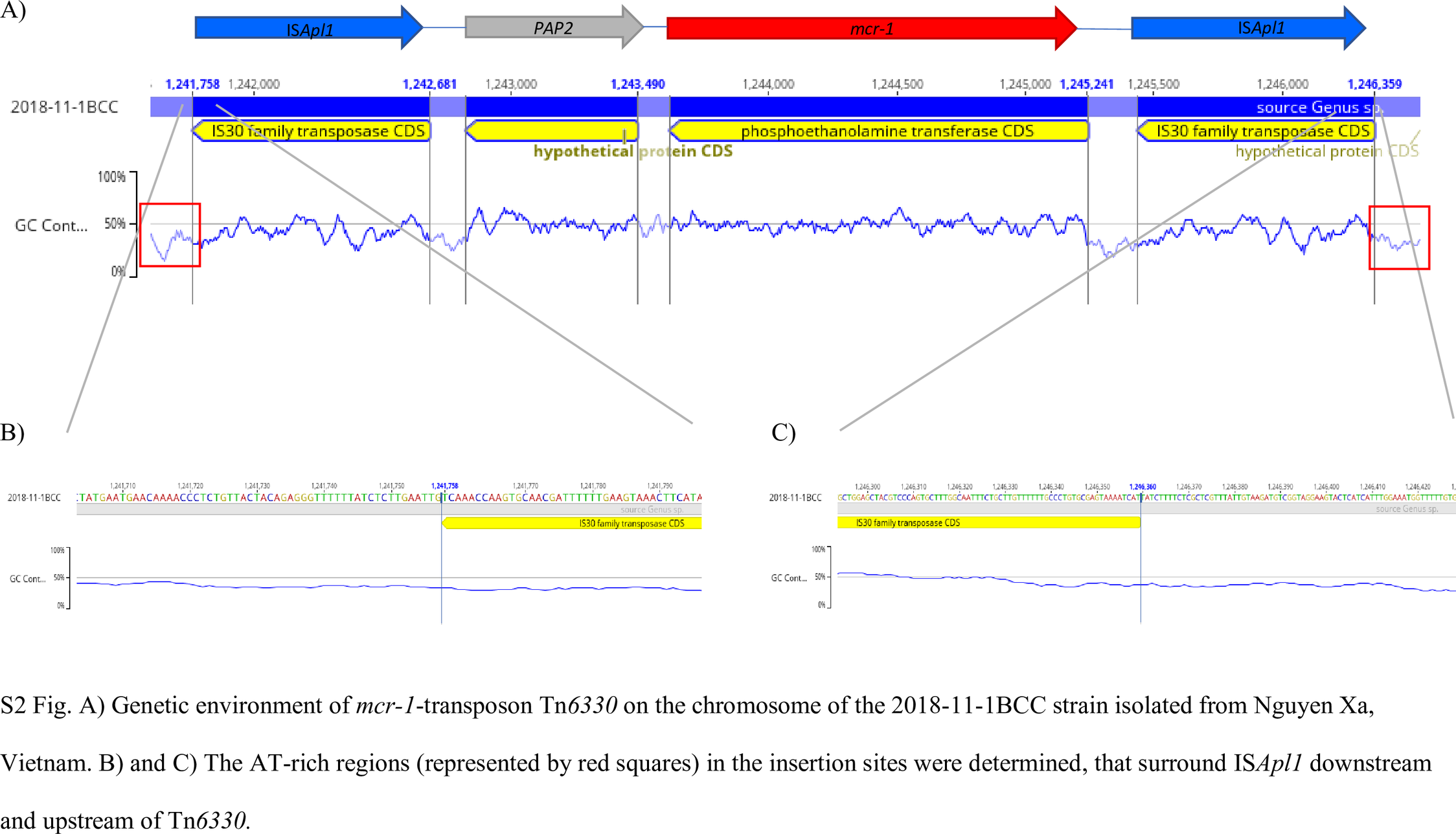

Supplement: S2 Fig — A) Genetic environment of mcr-1-transposon Tn6330 on the chromosome of the 2018-11-1BCC strain isolated from Nguyen Xa, Vietnam. B) and C) The AT-rich regions (represented by red squares), that surround ISApl1 downstream and upstream of Tn6330, in the insertion sites were determined. (TIF) [file pone.0293940.s002.tif]

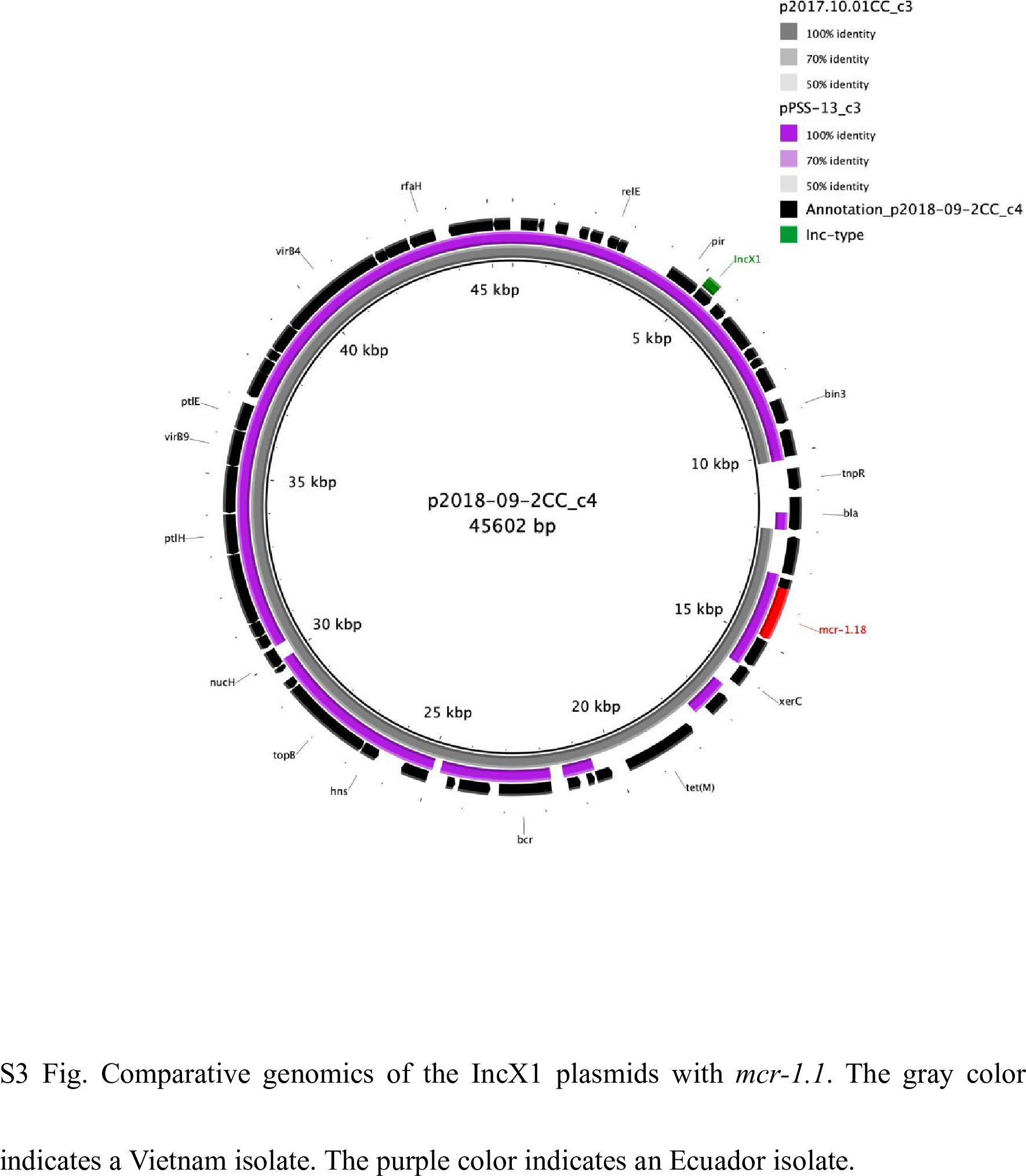

Supplement: S3 Fig — The purple color indicates an Ecuador isolate. (TIF) [file pone.0293940.s003.tif]

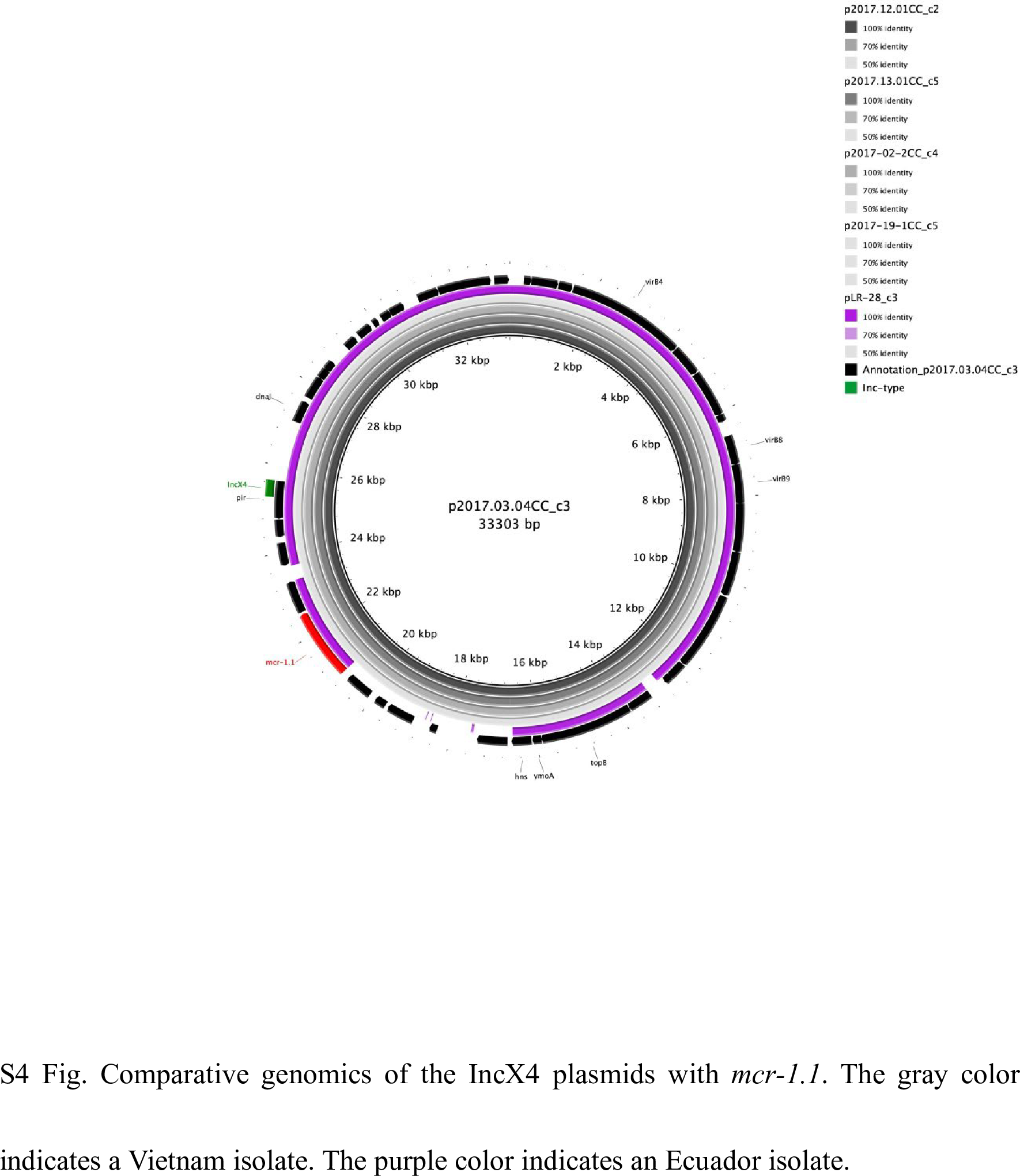

Supplement: S4 Fig — The purple color indicates an Ecuador isolate. (TIF) [file pone.0293940.s004.tif]

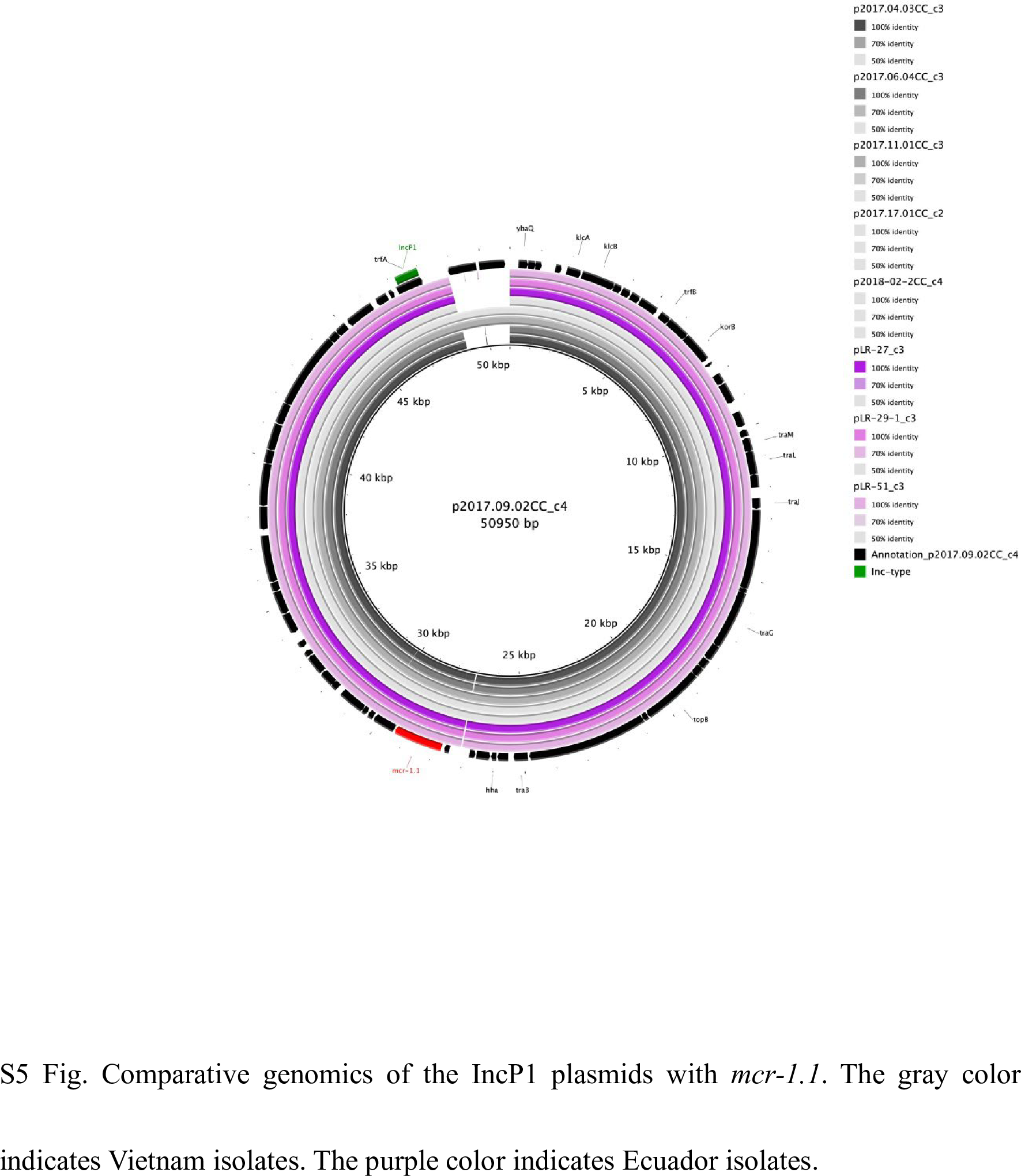

Supplement: S5 Fig — The purple color indicates Ecuador isolates. (TIF) [file pone.0293940.s005.tif]

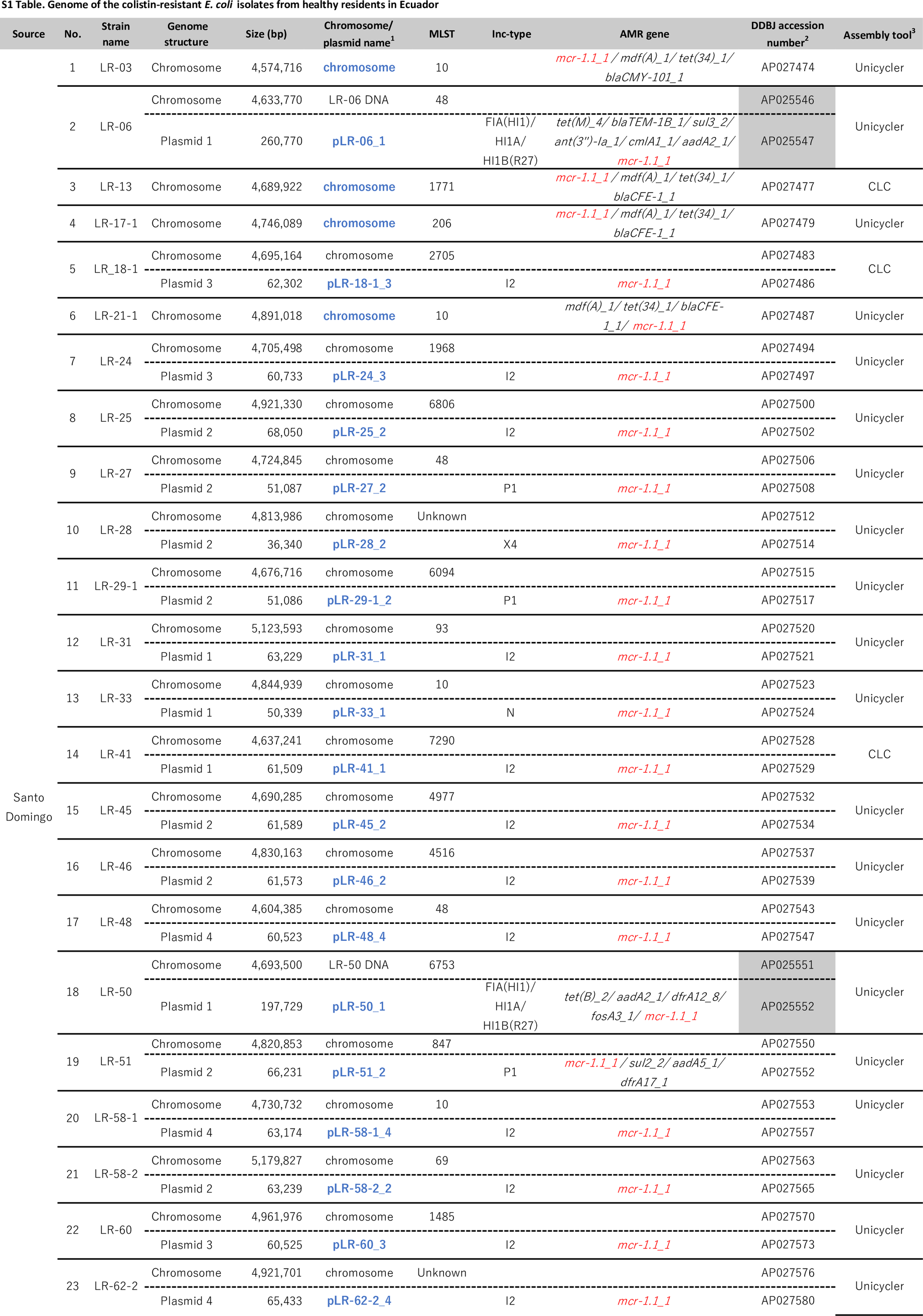

Supplement: S1 Table — (ZIP) [file pone.0293940.s006.zip › S1A_Table.tif]

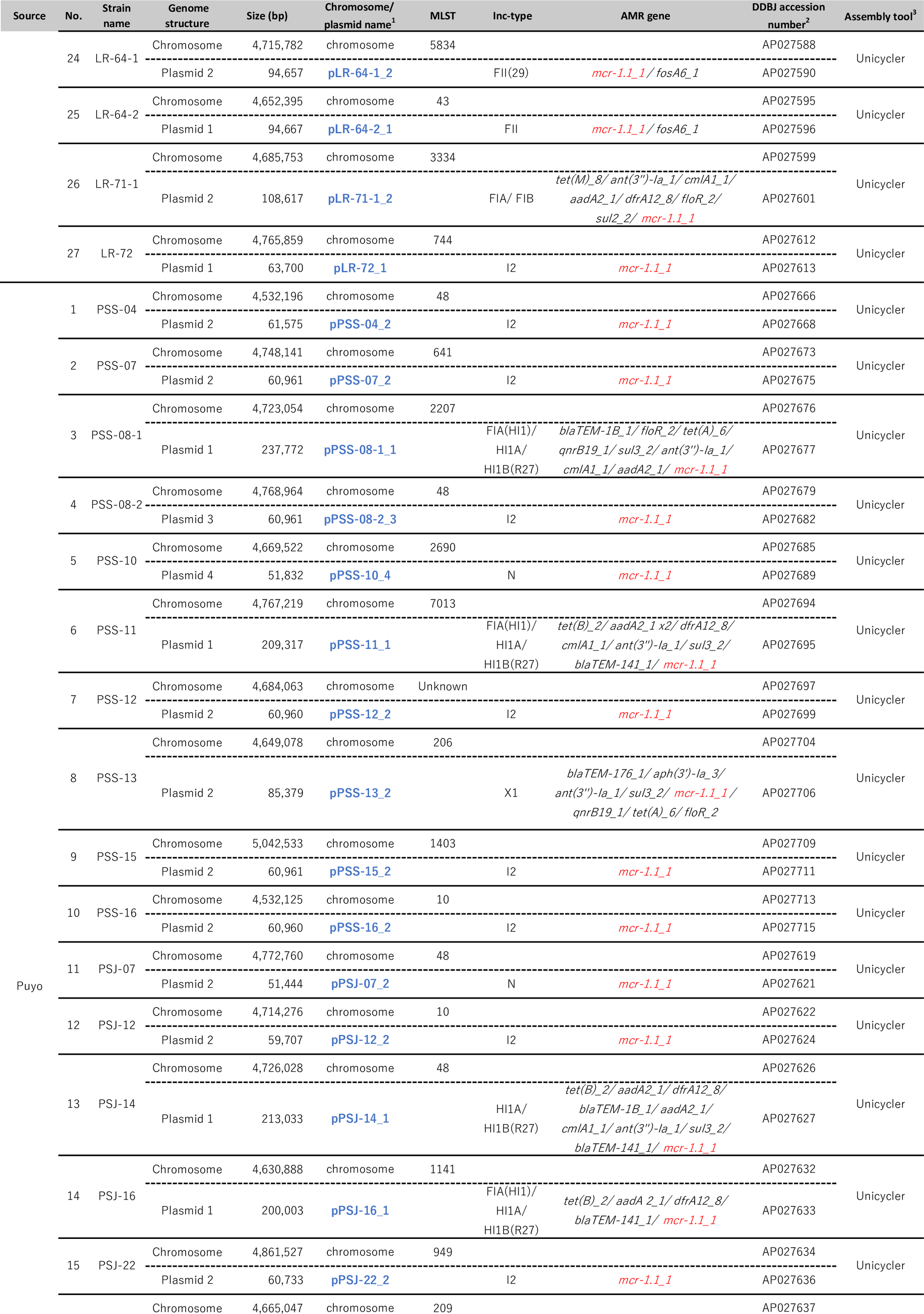

Supplement: S1 Table — (ZIP) [file pone.0293940.s006.zip › S1B_Table.tif]

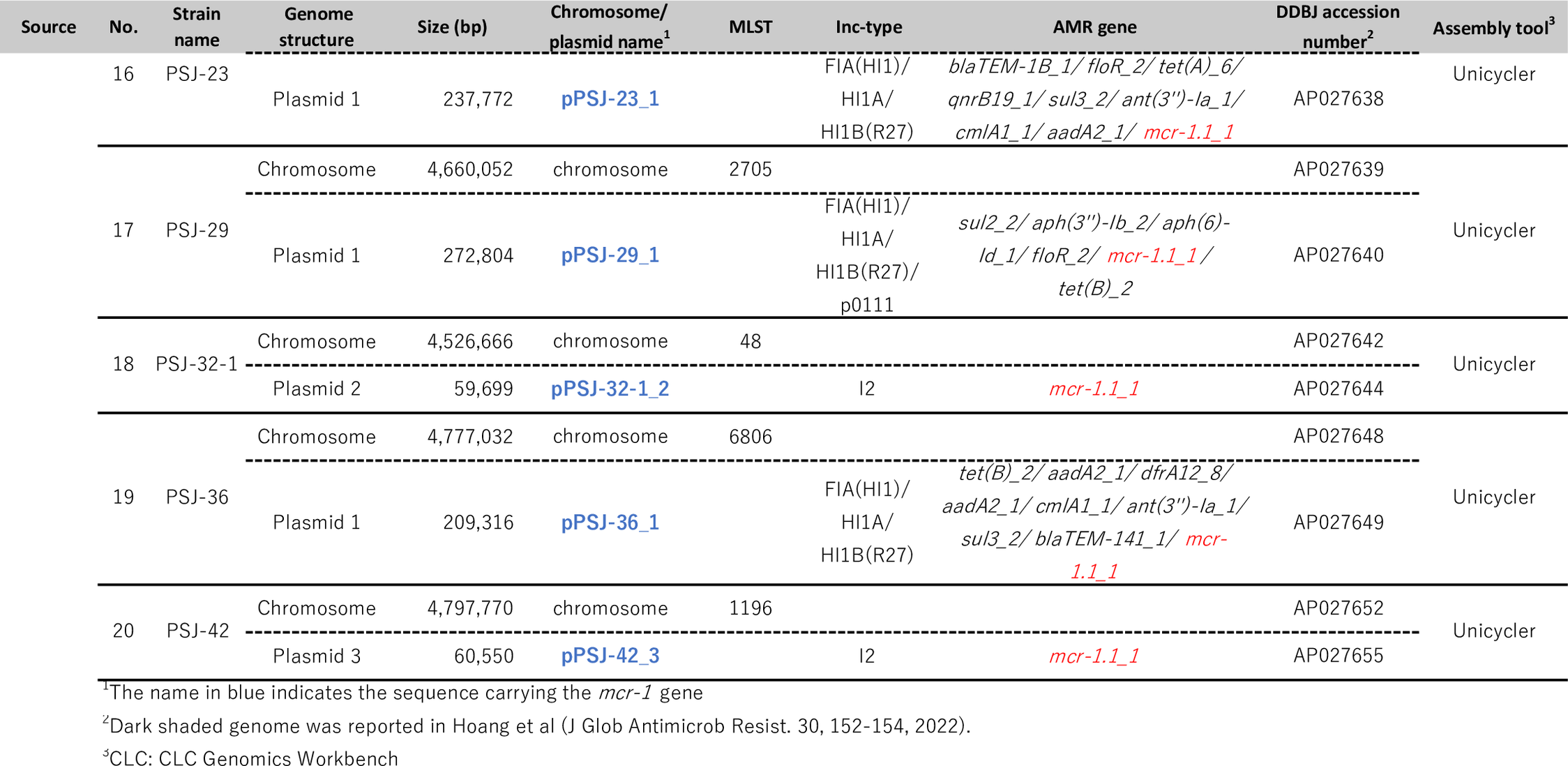

Supplement: S1 Table — (ZIP) [file pone.0293940.s006.zip › S1C_Table.tif]

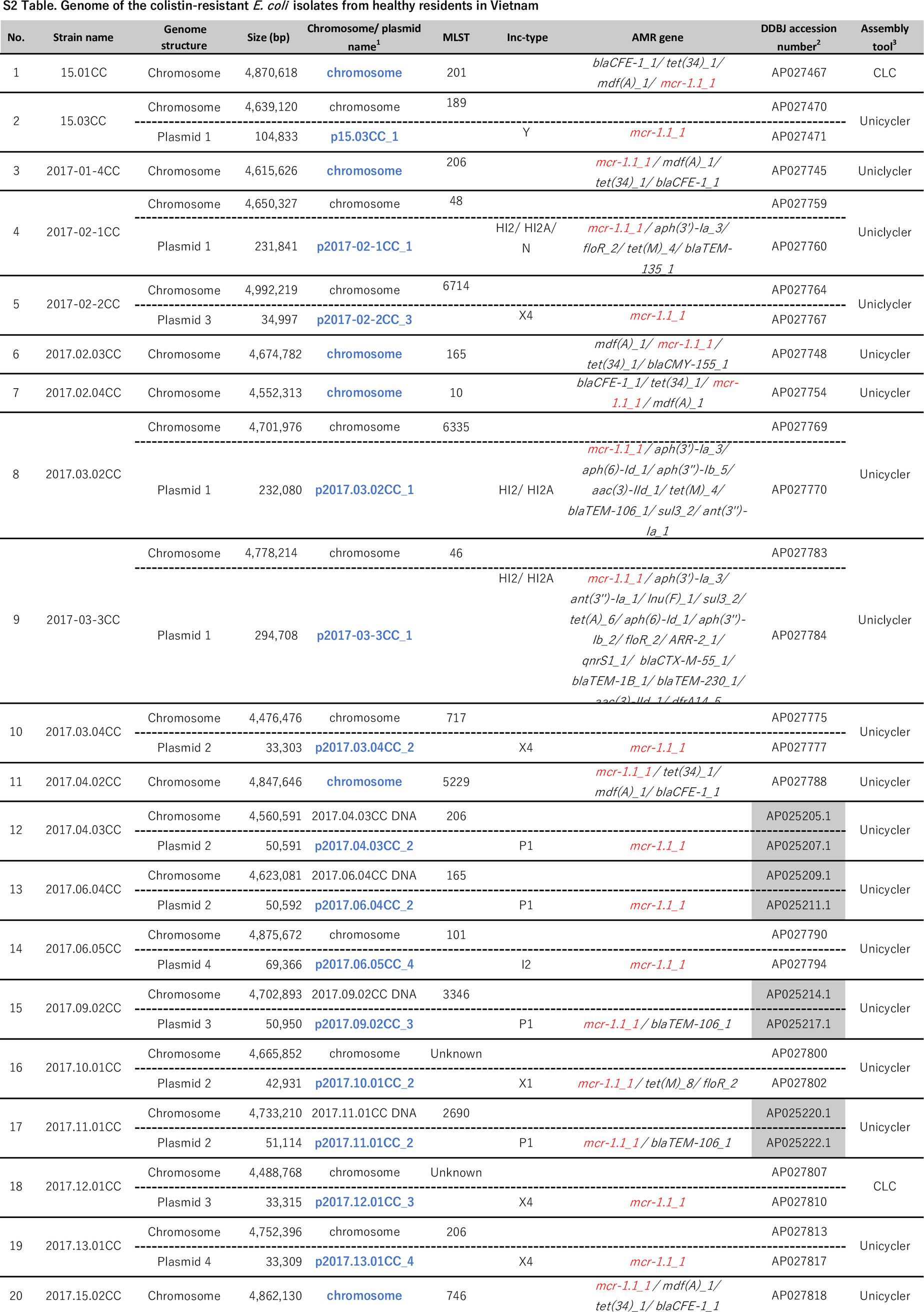

Supplement: S2 Table — (ZIP) [file pone.0293940.s007.zip › S2A_Table.tif]

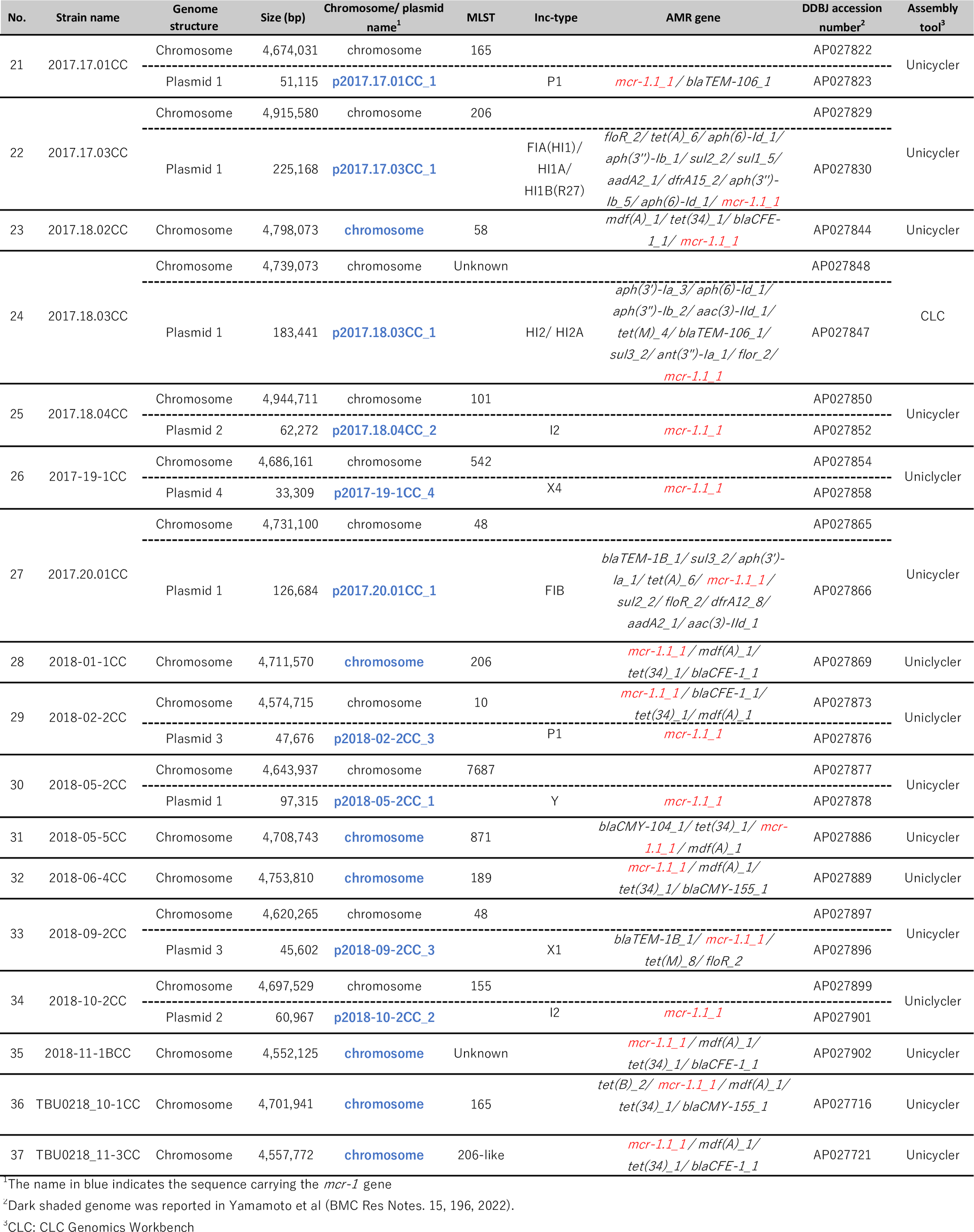

Supplement: S2 Table — (ZIP) [file pone.0293940.s007.zip › S2B_Table.tif]
